# Potential Predictors and Survival Analysis of the Relapse of HIV-Associated Cryptococcal Meningitis: A Retrospective Study

**DOI:** 10.3389/fmed.2021.626266

**Published:** 2021-05-10

**Authors:** Yao Li, Yanqiu Lu, Jingmin Nie, Min Liu, Jing Yuan, Yan Li, Huan Li, Yaokai Chen

**Affiliations:** ^1^Division of Infectious Diseases, Chongqing Public Health Medical Center, Chongqing, China; ^2^Department of Hygiene Toxicology, School of Public Health, Zunyi Medical University, Zunyi Guizhou, China; ^3^Division of Respiratory Geriatrics, Chongqing Public Health Medical Center, Chongqing, China

**Keywords:** HIV, cryptococcal meningitis, relapse, CD4+ T-cell count, timing of ART

## Abstract

**Objective:** We intend to investigate the relapse of HIV-associated cryptococcal meningitis (CM), assess potential predictors and conduct survival analysis, with a view to establishing a valid reference for the management of the relapse of CM.

**Method:** This is a retrospective study in Chinese patients with HIV-associated CM and those who experience relapse of CM. Baseline demographic, laboratory and clinical characteristics of patients with HIV-associated CM were collected. Predictors for relapse of HIV-associated CM were analyzed using univariate and multivariate logistic regression. Survival probability in relapse cases was determined by Kaplan-Meier survival curves.

**Results:** During the study period, 87 of 348 (25.0%) HIV patients experienced the relapse of CM. CD4+ T-cell counts, antiretroviral therapy (ART) status and the time from symptom onset to presentation were all statistically associated with the relapse of CM (*p* = 0.013, 0.018 and 0.042, respectively). The overall survival among 46 HIV CM relapse patients whose survival information were obtained, was 78.3%. The proportion of patients who died after antifungal treatment for CM was greater in those whose interval from symptom onset to presentation ≥4 weeks, compared with those <4 weeks (*p* = 0.0331).

**Conclusions:** In order to reduce the relapse of CM and increase the survival possibility of these patients, we can promote the importance of ART before CM occurs, emphasize timely consultation when any CM-associated clinical symptoms occurs, and individualized the timing of ART initiation according to indicators which can reflect the severity of CM.

## Introduction

Cryptococcal meningitis (CM) remains a major cause of mortality related to human immunodeficiency virus (HIV) infection (1), and the CM-associated mortality is as high as 15% globally in HIV-infected populations (2). Worryingly, even after appropriate antifungal treatment for CM, HIV-associated CM has a high relapse rate. A prospective observational study conducted in Cape Town, South Africa between 2007 and 2008, found that the relapse rate was 23.0% (69/300) among patients with HIV-associated CM, after induction treatment of CM (3). However, the relapse rate for HIV-associated CM has not been systematically reported in China, and this is especially significant because the currently recommended induction treatment regimen in China (4) is not consistent with that recommended by WHO (5).

Previous studies conducted before 2005 have reported that several risk factors are associated with the relapse of CM among HIV-infected patients. For instance, initial induction treatment with fluconazole monotherapy may be related to relapses of symptomatic CM, secondary to the development of fluconazole resistance, and immune reconstitution inflammatory syndrome (IRIS) (6). Not receiving flucytosine during the initial 2 weeks of primary treatment for cryptococcal disease is reported to be a factor associated with relapse of culture positive CM (7). In addition, receiving itraconazole as maintenance therapy for HIV-associated CM is more likely to be associated with culture-positive relapse, in comparison to receiving fluconazole as maintenance therapy (7). Discontinuation of maintenance therapy for CM is a risk factor for the relapse of CM if CD4+ T-cell counts are <100 cells/mL while receiving ART (8). The preceding studies analyzed risk factors for the relapse of HIV-associated CM from the perspective of induction and maintenance treatment. Additionally, the putative influence of individual demographic characteristics, laboratory parameters, and clinical characteristics of patients on whether relapse of HIV-associated CM occurs after a first episode of CM, is not clear. The mortality rate among relapse cases of CM, and the potential factors influencing survival in these patients have also not been investigated previously.

In the present study, we intend to comprehensively investigate the relapse of HIV-associated CM, assessing baseline characteristics of affected patients, potential risk factors for relapse, and conduct patient survival analysis, with a view to establishing a valid reference for the management of high-risk cases of CM relapse. In our study, relapse of CM was defined as the occurrence of more than one episode of CM, and which was also the reason for hospital readmission.

## Methods

### Study Design and Population

This is a retrospective study in patients with HIV-associated CM and those who experienced relapse of CM. HIV patients who were aged 18 years or older and had a definitive diagnosis of at least one episode of CM between August 2006 and June 2019, admitted to the Chongqing Public Health Medical Center, China, were enrolled in this study for evaluation. CM was diagnosed by isolation of cryptococcus from cerebrospinal fluid (CSF) cultures, positive CSF India ink staining and/or positive CSF cryptococcal antigen tests. This study was approved by the institutional review board of Chongqing Public Health Medical Center (No. 2019-003-02-KY). The institutional review board waived the requirement for written informed consent, since this study was entirely retrospective, and all patient data were anonymized.

Baseline demographic, laboratory, and clinical characteristics of 348 patients were collected, viz. age, sex, time from symptom onset to presentation, CD4+ T-cell count, HIV RNA viral load, cryptococcal antigen titer, CSF white-cell count, CSF protein level, ART status, timing of ART initiation, other concurrent neurological infections, maximum and minimum intracranial pressure, headache, fever, nausea, and vomiting.

### Assessment of the Risk of Relapse

Potential risk predictors were screened by retrieving related reports and general clinical experience. After excluding cases with missing data, the data of a total of 102 HIV patients who were diagnosed with CM, with complete medical records, were used for risk assessment via univariate and multivariate logistic regression. Among these 102 HIV patients, 49 were relapse cases and 53 were non-relapse cases. The following eight characteristics at baseline were considered to be associated with relapse of CM, taking into consideration clinical features, and relevant data from previous studies (6, 7, 9, 10): age, CD4+ T-cell counts, CSF white-cell count, CSF protein level, ART status, time from symptom onset to presentation, initial induction treatment with Amphotericin B (AmB), initial induction treatment with flucytosine.

### Survival Analysis

Of the original total of 348 patients with HIV-associated CM, a total of 87 patients experienced relapse of CM, and 46 of these patients were contactable by telephone. Thus, the survival status of 46 HIV-infected patients from inception until June 1st 2020 was obtained telephonically. Then, the cumulative probability of survival was assessed among these cases, and was compared between the time from symptom onset to presentation ≥4 weeks vs. <4 weeks, between CD4+ T-cell counts >20 cells/mm^3^ vs. ≤ 20 cells/mm^3^ and among those with earlier ART initiation (initiated within 4 weeks after diagnosis with CM) vs. deferred ART initiation (initiated 4 weeks or more after diagnosis with CM) vs. ART-experienced.

### Statistical Analysis

With regards to the characteristics of 348 HIV-infected patients, categorical variables were summarized using frequency and percentages, and continuous variables were summarized using median and interquartile ranges (IQR). Risk predictors for relapse of HIV-associated CM were analyzed using univariate logistic regression. Subsequently, all predictors were further subjected to multivariate stepwise forward logistic regression for further analysis.

Cumulative probability of survival in relapse cases was determined by Kaplan-Meier survival curves. All reported *p*-values are two-tailed, and a *p*-value of < 0.05 was considered statistically significant in all analyses. All analyses were performed using Stata v.14 (StataCorp, College Station, Texas, USA) and IBM-SPSS v.22 (Armonk, NY, USA).

## Results

### Characteristics of Patients

**Figure 1:**
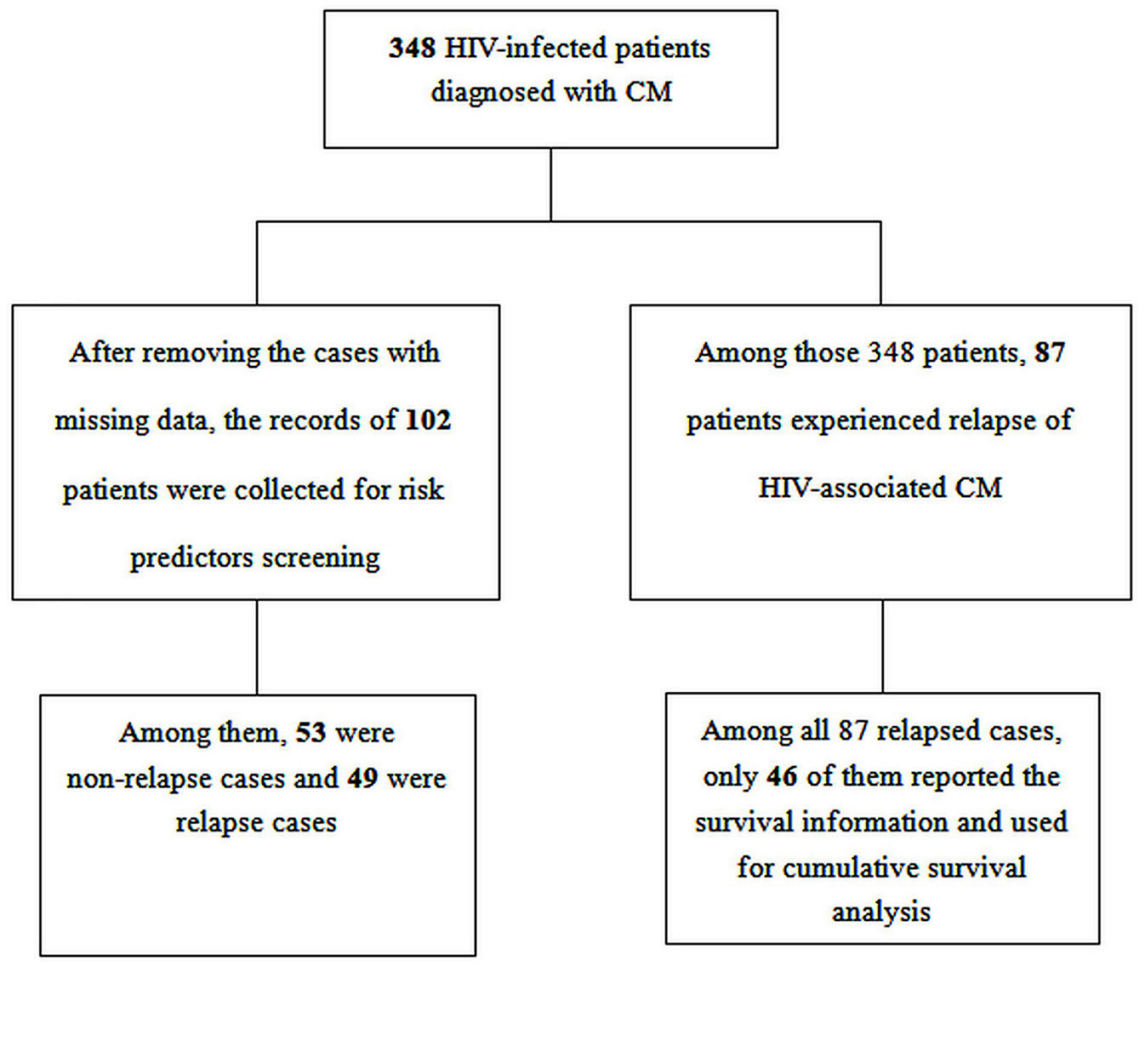
Study flow diagram.

During the study period, 348 HIV patients experienced their first episode of CM, and of these patients, 87 (25.0%, 87/348) experienced a relapse of CM, as shown in Figure 1. The median baseline HIV RNA viral load was 75,550 copies/mL (IQRs, 0–352,250) in all patients with HIV-associated CM, and 156,500 copies/mL (IQRs, 5,167–377,169) in those who experienced a relapse of CM. The median CSF white-cell count in patients with HIV-associated CM was 20 cells/mm^3^ (IQRs, 0.3–93), and that in those who experienced a relapse of CM was 0.05 cells/mm^3^ (IQRs, 0.3–93). The median CSF protein levels were 381.8 mg/dl (IQRs, 0.5–787.0) and 539.8 mg/dl (IQRs, 177.9–853.2) in patients with HIV-associated CM and in those who experienced relapse of CM, respectively. As displayed in Table 1, 11.5% (40/348) of patients with HIV-associated CM had other concurrent neurological infections (viz, tuberculous meningitis, toxoplasma encephalitis), and 25.3% (22/87) of patients who experienced the relapse of CM had other concurrent neurological infections.

**Table 1:** Baseline characteristics of patients with HIV-associated CM and cases of HIV-associated CM relapse.

**Characteristics**	**Patients with HIV-associated CM (*n* = 348)**	**Patients with relapse of HIV-associated CM (*n* = 87)**
	**Number (%) or Median [IQR]**	**Number (%) or Median [IQR]**
Median age (IQR)–years	46 [34–55.75]	42 [33–51]
Sex, male (%, n/n)	77.9% (271/348)	81.6% (71/87)
Median time from symptom onset to presentation (IQR)–days	15 [7-30]	10 [5-20]
Median CD4+ T-cell count (IQR)–cells/mm^3^	20 [7–40.5]	18 [7.25–36.75]
Median HIV RNA level (IQR)–copies/ml	75550 [0–352250]	156500 [5167–377169]
Median AST (IQR)	27 [17-40]	25 [17–35.5]
Median ALT (IQR)	24 [15-39]	27 [14.25–37]
Median TBIL (IQR)	10.8 [7.9–15.6]	9.3 [7.1–13.9]
Median DBIL (IQR)	4.5 [3.1–66]	4.2 [2.9–5.9]
Median IDBIL (IQR)	6.3 [4.5–9.1]	5.6 [4.1–7.3]
Median CREA (IQR)	60.7 [49.7–73.7]	58.0 [47.8–73.9]
Median HGB (IQR)	112.0 [97–127]	110.0 [96.0–124.5]
Median Cryptococcal antigen titer (IQR)	0 [0–80]	0 [0–80]
Median CSF white-cell count (IQR)–cells/mm^3^	20 [0.3–93]	0.05 [0–16.5]
Median CSF protein level (IQR)–mg/dl	381.8 [0.5–787.0]	539.8 [177.9–853.2]
ART-experienced patients (%, n/n)	38.2% (57/149)	40.7% (35/86)
Median ART initial time (IQR)–days	23 [0–34.5]	17 [0–33.5]
Other concurrent neurological infections (%, n/n)	11.5% (40/348)	25.3% (22/87)
Median maximum intracranial pressure at first admission (IQR)–mm H_2_O	300 [200–400]	230 [0–330]
Median minimum intracranial pressure at first admission (IQR)–mm H_2_O	140 [90–200]	130 [80–181.2]
**Clinical symptoms:**		
Headache (%, n/n)	73.3% (255/348)	78.2% (68/87)
Fever (%, n/n)	57.8% (201/348)	54.0% (47/87)
Nausea (%, n/n)	29.0% (101/348)	21.8% (19/87)
Vomiting (%, n/n)	36.2% (126/348)	29.9% (26/87)

*IQR, inter quartile range; AST, aspartate aminotransferase; ALT, alanine aminotransferase; TBIL, total bilirubin; DBIL, direct bilirubin; IDBIL, indirect bilirubin; CREA, creatinine; HGB, hemoglobin*.

### Predictors of Relapse of HIV-Associated CM

After excluding case records of patients with missing data, the medical records of the remaining 102 patients were analyzed for risk predictor analysis. The CD4+ T-cell count ≤ 20 cells/mm^3^ group, when compared with the CD4+ T-cell count >20 cells/mm^3^ group, had a significant higher risk of relapse of HIV-associated CM (OR with 95%CI: 2.614, 1.161–5.886, *p* = 0.020). As shown in Table 2, there were no significant differences in timing of ART initiation among ART-naïve patients between the non-relapse group and the relapse group (*p* = 0.069). In contrast, compared with the ART-naive patients who were initiated ART <4 weeks after antifungal treatment, it was more likely for patients in the ART-experienced group to suffer relapse of HIV-associated CM (OR with 95%CI: 2.838, 1.048–7.686, *p* = 0.020). However, among the 19 ART-experienced patients with CM relapse, we found that over 57.9% (11/19) reported either drug withdrawal without appropriate medical guidance, irregular medication consumption, or poor compliance with ART prescription. As for induction therapy, there was no significant difference (*p* = 0.175) in completion of induction therapy between the non-relapse group (*n* = 40) and the relapse group (*n* = 42), as shown in Supplementary Table 1. Similarly, no statistical difference was found among patients who initiated consolidation therapy (*p* = 0.912) between the non-relapse group (*n* = 36) and the relapse group (*n* = 30).

**Table 2:** Univariate and multivariate regression assessment of relapse of CM (*n* = 102).

**Characteristics**	**Numbers of non-relapse cases (*n* = 53)**	**Numbers of relapse cases (*n* = 49)**	**Univariate analysis**		**Multivariate analysis**
			**OR (95%CI)**	***p-*value**	***p-*value**
**Age**					
<60 years	44	38			
≥60 years	9	11	1.415 (0.530–3.779)	0.488	–
**Baseline CD4 cell counts**					
>20 cells/mm^3^	37	23			
≤ 20 cells/mm^3^	16	26	2.614 (1.161–5.886)	0.020	0.013
**CSF white-cell counts**					
≤ 20 cells/mm^3^	22	26			–
>20 cells/mm^3^	31	23	0.628 (0.287–1.374)	0.244	
**CSF protein levels**					
≤ 500 mg/dl	17	26			
>500 mg/dl	36	23	0.813 (0.359–1.844)	0.621	–
**ART status**					
Early ART	23	11		0.084	
Deferred ART	16	19	2.483 (0.933–6.609)	0.069	
ART-experienced	14	19	2.838 (1.048–7.686)	0.040	0.018
**Time from symptom onset to presentation**					
≥4 weeks	16	9			
<4 weeks	37	40	1.922 (0.758–4.876)	0.169	0.042
**Induction therapy includes AmB**					
No	11	7			–
Yes	42	42	1.571 (0.556, 4.444)	0.394	
**Induction therapy includes 5-FC**					
No	32	22			
Yes	21	27	1.870 (0.851, 4.110)	0.119	–

*Early ART: <4 weeks after starting antifungal treatment*.

*Deferred ART: 4 weeks or more after starting antifungal treatment*.

*ART-experienced: Initial ART before starting antifungal treatment*.

After accounting for the effects of confounding factors by utilizing multivariate analysis, CD4+ T-cell counts, ART status, and the time from symptom onset to presentation at baseline were all statistically associated with the relapse of HIV-associated CM (*p* = 0.013, 0.018, and 0.042, respectively), as listed in Table 2.

### Survival Analysis

**Figure 2:**
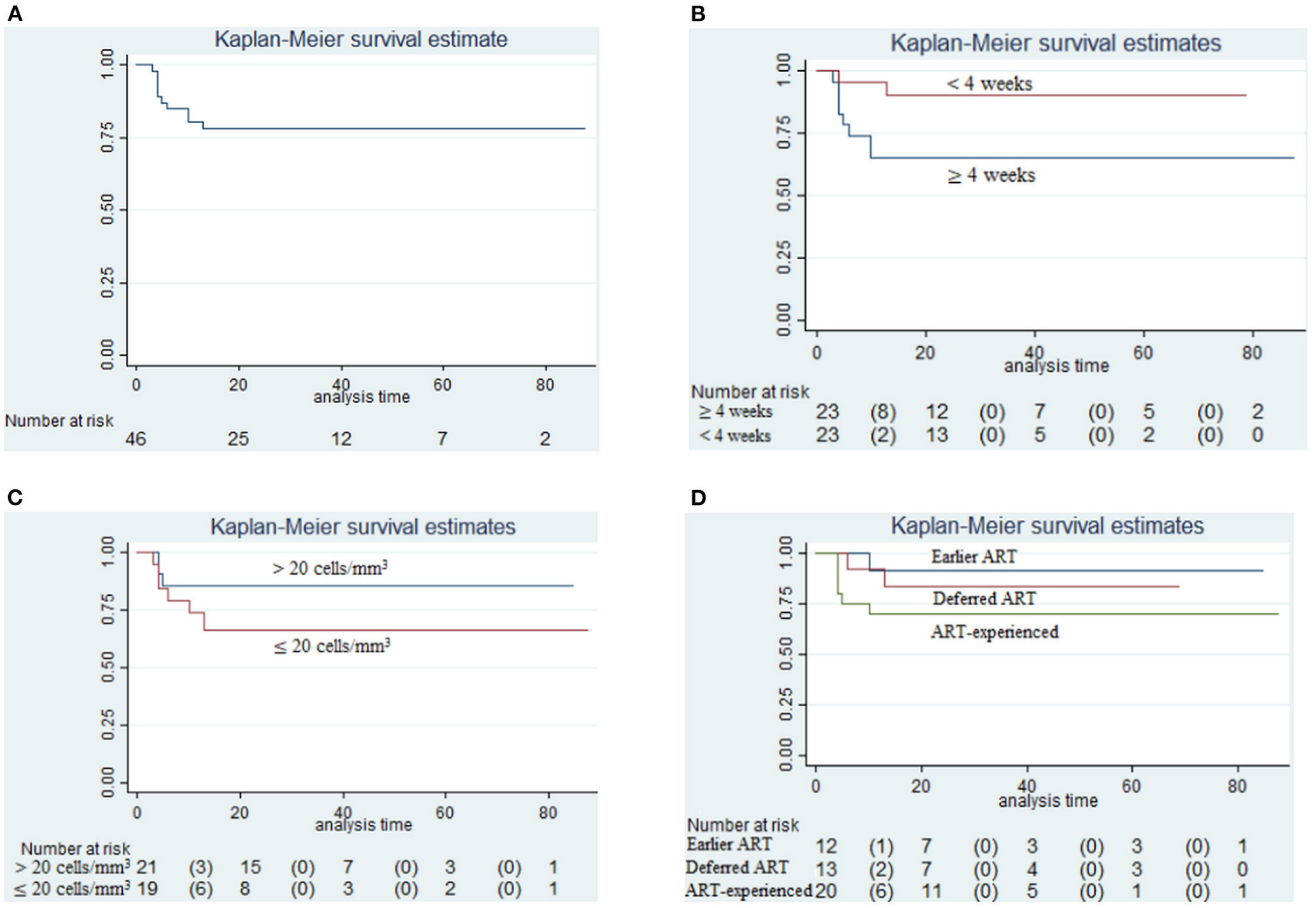
Survival analysis. **(A)** Cumulative probability of survival in relapse cases relative to initiation of antifungal treatment (until June 1st, 2020); **(B)** Survival analysis in those between the time from symptom onset to presentation ≥4 weeks and <4 weeks (*p* = 0.0331); **(C)** Survival analysis in those having CD4+ T-cell counts >20 cells/mm^3^ and CD4+ T-cell counts ≤ 20 cells/mm^3^ (*p* = 0.1849); **(D)** Survival analysis in those prescribed earlier ART initiation (initial ART within 4 weeks after antifungal treatment) and deferred ART initiation (initial ART ≥4 weeks after antifungal treatment) and those already on ART (initiation of ART before antifungal treatment) (*p* = 0.2632).

The overall survival among 46 HIV CM relapse patients was nearly 75% from the time of antifungal treatment initiation of the earliest case until June 1st 2020 (0–88 months), as shown in Figure 2A. The proportion of patients who died after the 10th month of antifungal treatment for CM was greater in those whose interval from symptom onset to presentation was ≥4 weeks, compared with those whose interval from symptom onset to presentation was <4 weeks (8 of 23 [35%] vs. 2 of 23 [9%]; *p* = 0.0331), as shown in Figure 2B. However, the difference in all-cause mortality between the group whose CD4+ T-cell counts were >20 cells/mm^3^, and the group whose CD4+ T-cell counts were ≤ 20 cells/mm^3^, was not found to be significant from 0 to 88 months (*p* = 0.1849), as shown in Figure 2C. During the study period, 1 of 12 patients (8%) in the earlier ART initiation group (initial ART within 4 weeks after antifungal treatment), 2 of 13 patients (15%) in the deferred ART group (initial ART ≥4 weeks after antifungal treatment), and 6 of 20 patients (30%) in the ART-experienced group died (initial ART before antifungal treatment). All-cause mortality amongst these three groups did not differ significantly (*p* = 0.2632), as shown in Figure 2D.

## Discussion

The relapse rate of CM was high in the present study, at 25.0% (87/348). When taking into account onward referral of CM patients from this center, and outpatient death, the relapse rate would be even greater. Previous studies have reported that the annual relapse rates for cryptococcal disease in developed countries, where Amphotericin (AmB)-based regimens are the standard initial therapy (6), is <5% during maintenance fluconazole treatment (11, 12). However, in our study, the relapse rate was 50.0% (42/84) among patients who started therapy with AmB-based regimens. In addition, in contrast to the short phase induction regimen for cryptococcal disease recommended by current WHO guidelines (1 week of AmB and flucytosine, followed by 1 week of fluconazole) (5), the Chinese Academy of Medical Sciences recommends at least 4 weeks of AmB combined with flucytosine for the treatment of HIV-associated CM in China (4) as the induction phase. It is, therefore, quite pertinent and important to attempt to identify valid reasons for this high relapse rate, and to strive to effectively manage and mitigate this unacceptably high risk by developing appropriate management protocols after diligent investigation.

Previous studies have reported that several risks factors are related to the relapse of CM among HIV-infected patients. Firstly, the choice of fluconazole monotherapy as induction therapy, has a higher mortality risk when compared with AmB-based regimens (13). Secondly, not receiving flucytosine during the initial 2 weeks of primary treatment is a specific factor associated with the relapse of cryptococcal disease (relative risk = 5.88; *p* = 0.04) (7). Thirdly, among HIV-infected patients who had been successfully treated (achieved negative-culture of CSF) for a first episode of CM, the culture-positive relapse rate was higher in the itraconazole group than in the fluconazole group (23% vs. 4%, *p* = 0.006) (7). In addition, it has been reported in a prospective observational study that 43.5% (30/69) of relapses episodes occur in patients not taking fluconazole secondary prophylaxis (3). The other common reason for the relapse of cryptococcosis during the maintenance treatment phase is inadequate treatment with the primary antifungal drug(s) and/or non-adherence to secondary prophylaxis (14). Additionally, in a previously discussed prospective observational study, 44.9% (31/69) of relapse episodes were due to paradoxical IRIS (3). A retrospective study conducted in Taiwan suggested that an initial CD4+ T-cell count of <20 cell/mm^3^ is a risk factor for relapse of CM or death in HIV-infected patients (9), which is consistent with the results of our study. In summary, previous studies have suggested that inadequate induction and maintenance therapy, poor compliance during the maintenance treatment phase, inappropriate ART initiation timing, and low baseline CD4+ T-cell counts may all be associated with relapse of HIV-associated CM.

A prospective observational study conducted in Cape Town, South Africa, found that CM relapse accounted for 23.0% (69/300) of all cases of HIV-associated CM (3), which is consistent with the 25% (87/348) found in the present study. A previous randomized controlled trial showed that the relapse of CM is associated with initial timing of ART among HIV-infected patients (10). Specifically, in the preceding study, the relapse rate for HIV-associated CM was 2% (95%CI, 1–7%) in those initiating ART at 2–4 weeks after initiating antifungals, and 9% (95%CI, 4–16%) in those initiating ART at 4–6 weeks after initiating antifungals, *p* = 0.06 (10). A systematic review compared the outcomes of early initiation of ART (<4 weeks after starting antifungal treatment) vs. delayed initiation of ART (4 weeks or more after starting antifungal treatment) in HIV-infected patients with CM, and the results indicates that early ART initiation may reduce the relapse of CM compared to delayed ART initiation (RR with 95%CI: 0.27, 0.07–1.04; low-certainty evidence) (15). In the present study, the number of CM relapse cases in the earlier ART initiation group (≤4 weeks) and in the delayed ART initiation group (>4 weeks) were 11 and 19, respectively; however, the difference between the two groups was not statistically significant (*p* = 0.069). However, another randomized controlled trial conducted by Boulware et al. (10), suggested that for patients with CSF white-cell counts <5 cells/mm^3^ at baseline, the 26-week mortality rate in the early ART initiation group (1-two weeks after diagnosis) was significant higher than in the delayed ART initiation group (5 weeks after diagnosis) (Hazard ratio with 95%CI, 1.73, 1.06–2.82, *p* = 0.03). Additionally, among patients who experienced a relapse of CM in the present study, 29.9% (26/87) had a CSF white-cell count <5 cells/mm^3^, and had a higher risk of death if ART was initiated within 4 weeks. Thus, in order to improve survival in patients who have a baseline CSF white-cell count <5 cells/mm^3^, clinicians may wish to consider deferring ART initiation for at least 4 weeks after the diagnosis of CM. Moreover, in the present study, patients who had initiated ART before diagnosis with CM experienced an unexpected high probability of relapse, compared with those patients who initiated ART within 4 weeks of diagnosis of CM (19 vs. 11). However, more than half of those 19 patients (11/19) reported drug withdrawal without appropriate medical guidance, irregular medication consumption, or poor compliance with ART prescription, which suggests that poor compliance of ART increases the risk of relapse of CM in HIV-infected patients. The results of survival analysis showed that the patient all-cause mortality rate was high if the interval from symptom onset to presentation was over 4 weeks, which indicates that failure to treat expeditiously is not only more likely to cause relapse of HIV-associated CM, but also more likely to increase the risk of death from it.

There are some limitations in this study. Firstly, this is all about Chinese HIV patients, and some patients who did not report a relapse episode of CM were either discharged from care and did not present for follow-up, or refused further care. Nevertheless, these patients have a high probability of subsequent relapse of CM. Thus, our study underestimates the relapse rate of CM, and introduces a degree of bias to the results. Secondly, because it is clinically difficult to definitively and precisely distinguish between relapse of CM and paradoxical CM-IRIS, we have not evaluated the association between IRIS and relapse. Thirdly, due to the present investigation being a retrospective study, the prescribed courses of antifungal treatment for CM was not fixed or standardized, and only 46 of 87 patients reported survival information. Additionally, some patients were still undergoing induction antifungal treatment when the second episode of CM occurred. Also, some patients were yet to initiate secondary prophylaxis during the period of our study, and there was no relevant data regarding compliance with secondary prophylaxis. Therefore, the association between secondary prophylaxis and CM relapse has not been assessed in the present study. However, secondary prophylaxis has been recommended by WHO guidelines (5), and studies have clearly shown that failure to prescribe appropriate secondary prophylaxis increases the incidence of relapse (16–19).

## Conclusions

In order to reduce the relapse of HIV-associated CM and increase the survival possibility of patients who experienced the relapse of CM, we propose the following strategies for the management of high-risk cases of CM relapse: (1). For HIV positive patients who have not yet developed any opportunistic infections, the importance of strict and assiduous ART compliance by patients should be repeatedly emphasized and positively reinforced at ART initiation and during follow-up; (2). During follow-up, health clinic workers should remind HIV-infected patients to seek medical treatment urgently (within 4 weeks) if headaches, fever, vomiting, and other CNS-related symptoms occur, the sooner the better; (3). If the baseline CSF white-cell counts of ART-naïve patients are <5 cells/mm^3^, it is prudent to initiate ART 4 weeks after initiation of antifungals.

## Data Availability Statement

The raw data supporting the conclusions of this article will be made available by the authors, without undue reservation.

## Ethics Statement

The studies involving human participants were reviewed and approved by the institutional review board of Chongqing Public Health Medical Center. The patients/participants provided their written informed consent to participate in this study.

## Author Contributions

YaoL, HL, and YC conceived and designed the protocol and study. JN, ML, and JY identified cases for eligibility. YaoL and YanqL extracted data of included cases. YaoL performed the data analysis, with assistance from YanL, HL, and YC. HL and YC drafted the article for important content. All authors read and approved the final manuscript.

## Conflict of Interest

The authors declare that the research was conducted in the absence of any commercial or financial relationships that could be construed as a potential conflict of interest.
