# Supplementary material for: Potential Predictors and Survival Analysis of the Relapse of HIV-Associated Cryptococcal Meningitis: A Retrospective Study
Source: Front Med (Lausanne). 2021 May 10;8:626266. doi: 10.3389/fmed.2021.626266 (PMC8141581; doi:10.3389/fmed.2021.626266)
Supplement: Supplementary file 1 [file Table_1.DOC]

**Table S1. Univariate regression assessment of relapse of CM (N=102)**

| **Characteristics** | **Numbers of non-relapse cases (N=53)** | **Numbers of relapse cases (N=49)** | **Univariate analysis** | |
| --- | --- | --- | --- | --- |
|  |  |  | **OR (95%CI)** | ***p* value** |
| **Completion of induction therapy**  No;  Yes;  Unclear | 12;  40;  1 | 6;  42;  1 | 2.100 (0.719-6.131);  2.000 (0.106-37.830) | 0.398;  0.175;  0.644 |
| **Initiation of consolidation therapy**  No;  Yes;  Unclear | 16;  36;  1 | 14;  30;  5 | 0.952 (0.401-2.263);  5.714 (0.594-54.962) | 0.279  0.912;  0.131 |
